# Live Cell Imaging Demonstrates Multiple Routes Toward a STAT1 Gain-of-Function Phenotype

**DOI:** 10.3389/fimmu.2020.01114

**Published:** 2020-06-09

**Authors:** Simone Giovannozzi, Veerle Lemmens, Jelle Hendrix, Rik Gijsbers, Rik Schrijvers

**Affiliations:** ^1^Department of Microbiology, Immunology and Transplantation, Allergy and Clinical Immunology Research Group, KU Leuven, Leuven, Belgium; ^2^Laboratory for Viral Vector Technology and Gene Therapy, Department of Pharmaceutical and Pharmacological Sciences, KU Leuven, Leuven, Belgium; ^3^Dynamic Bioimaging Lab, Advanced Optical Microscopy Center and Biomedical Research Institute, Hasselt University, Hasselt, Belgium; ^4^Molecular Imaging and Photonics Division, Chemistry Department, KU Leuven, Leuven, Belgium; ^5^Leuven Viral Vector Core, KU Leuven, Leuven, Belgium; ^6^Department of Microbiology, Immunology and Transplantation, Immunogenetics Research Group, KU Leuven, Leuven, Belgium

**Keywords:** STAT1, gain of function, live cell imaging, molecular mechanism, hyperphosphorylation, hypermorphic mutations

## Abstract

Signal transducer and activator of transcription 1 (STAT1) gain-of-function (GOF) mutations result in a primary immunodeficiency (PID) characterized typically by chronic mucocutaneous candidiasis (CMC), but a wider phenotypic range is reported and remains unexplained from a pathophysiological point-of-view. We hypothesized that different STAT1 GOF mutations may result in distinct molecular mechanisms, possibly explaining the variable phenotypes observed in patients. We selected STAT1 GOF mutants (R274W, R321S, T419R, and N574I) that are spread over the protein and studied their dynamic behavior *in vitro* in U3A and HeLa cell lines. All GOF mutants showed increased STAT1 phosphorylation compared to STAT1 WT. Real-time imaging demonstrated three underlying mechanisms for STAT1 GOF: (i) R274W showed a faster nuclear accumulation, (ii) both R321S and N574I showed a reduced nuclear mobility and slower dephosphorylation, whereas (iii) T419R was near-immobile in the nucleus, potentially due to enhanced binding to chromatin.

## Introduction

Autosomal dominant (AD) signal transducer and activator of transcription 1 (STAT1) gain-of-function (GOF) mutations result in a primary immunodeficiency (PID) characterized by chronic mucocutaneous candidiasis (CMC), recidivating respiratory infections, autoimmunity, and vascular anomalies. First described in 2011 (1, 2), to date 82 different mutations have been reported in more than 274 patients (2–16). A hallmark is increased susceptibility for fungal (*Candida*) infections, leading to CMC in 98% of the patients (8). Interestingly, additional phenotypes including John Cunningham (JC)-virus induced progressive multifocal leukoencephalopathy (11), Orf infection (6), Immunodysregulation polyendocrinopathy enteropathy X-linked (IPEX)-like syndromes with CMC (4, 15), and a combined immunodeficiency (CID) without CMC (16) have been associated with STAT1 GOF mutations, but remain unexplained from a pathophysiological point-of-view and therefore require further investigation. We hypothesized that different STAT1 GOF mutations may result in distinct molecular mechanisms, possibly explaining the variable phenotypes observed in patients.

Current treatment consists of chronic antifungal therapy for CMC, and episodic antibiotics and antivirotics, as well as immunosuppressive treatment in case of auto-immune manifestation. Granulocyte colony-stimulating factor treatment was reported to have low efficacy, with a single patient out of eight responding positively to the treatment (8, 17). Recently, short-term use of ruxolitinib, a Janus kinase (JAK) 1/2 inhibitor, has been demonstrated to be effective *in vitro* and *in vivo* in some STAT1 GOF patients with alopecia (18) or debilitating CMC (19–21). However, results have been conflicting, since not all patients responded to the treatment (12, 20), and effects dampened for unknown reasons in one patient, after the treatment was interrupted and later on restarted (13). Moreover, the concern remains that inhibition of the JAK/STAT1 pathway might tip the GOF over to a loss of function (LOF) phenotype with increased susceptibility for viral and mycobacterial infection [analogous to the already described autosomal recessive (AR) STAT1 deficiency (22), AR partial STAT1 (23) deficiency and AD STAT1 LOF (24)]. Finally, allogenic hematopoietic stem cell transplantation (HSCT) has been performed for some STAT1 GOF patients but associated with a poor outcome [2/6 survivors after 2y (8, 25) and 6/15 survivors in a more recent multi-center HSCT study for STAT1 GOF (26)].

**Figure 1:**
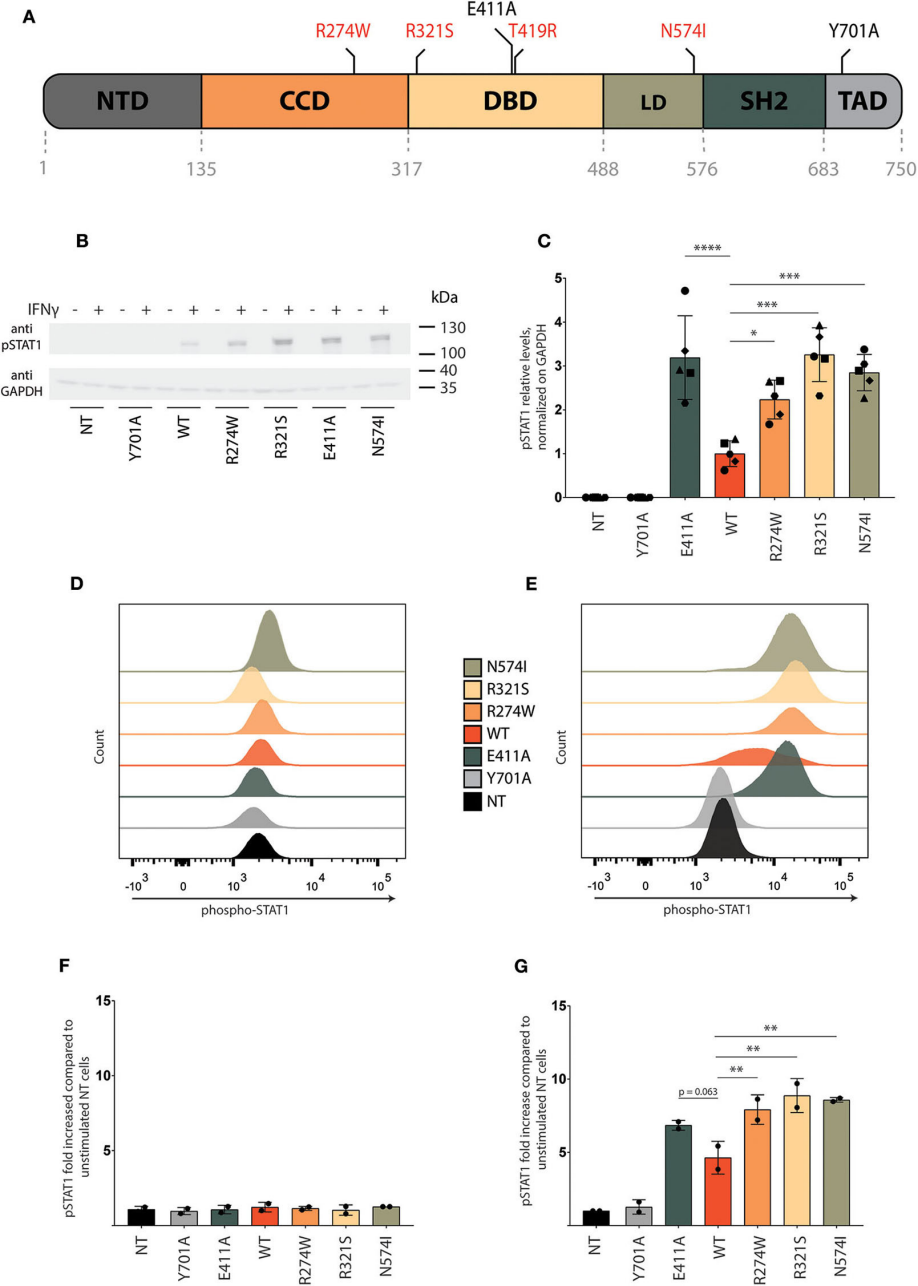
Characterization of STAT1 GOF mutant cell lines. **(A)** Representative scheme of STAT1 and its domains: N-terminal domain (NTD), coiled-coil domain (CCD), DNA binding domain (DBD), linker domain (LD), SRC homology domain (SH2), and transactivation domain (TAD). Positions of the GOF mutations (R274W, R321S, T419R, N574I) analyzed in this study are indicated in red, as well as the position of the controls in black (E411A, Y701A). **(B)** Representative Western blot of U3A cell lysate stained with anti-pSTAT1 and anti-GAPDH. Cells were stimulated for 1 h with IFNγ (1 U/μl) or left unstimulated. Five independent experiments were pooled together. **(C)** Quantification of Western blot pSTAT1 levels of U3A cells stimulated for 1 h with IFNγ (1 U/μl) from five independent experiments. **(D,E)** Representative pSTAT1 levels in the respective stable U3A cells stimulated for 1 h with IFNγ **(E)** or left unstimulated **(D)** measured via flow cytometry. **(F,G)** Quantification of pSTAT1 levels measured via flow cytometry of U3A cells stimulated for 1 h with IFNγ (1U/μl) **(G)** or left unstimulated **(F)**, for two independent experiments. Data are represented in bar graphs as mean ± standard deviation. Statistical analysis: One-way ANOVA with Dunnett's *post-hoc* test. *P < 0.05; **P < 0.01; ***P < 0.001; ****P < 0.0001.

STAT1 is a transcription factor that plays a pivotal role in the immune response and the Interferon (IFN) signaling pathway, modulating diverse cellular processes including immunity, proliferation, differentiation, and cell death (27–29). STAT1 is a member of the STAT family of proteins comprising other six members. STAT proteins are highly conserved and all present six domains: an N-terminal domain (NTD), a coiled-coil domain (CCD), a DNA binding domain (DBD), a linker domain (LD), a SCR2 homology domain (SH2), and a trans-activation domain (TAD), as depicted in Figure 1A. STAT1 is present in the cytoplasm of unstimulated cells as inactive, antiparallel homodimer (30, 31). Upon interferon gamma (IFNγ) stimulation, JAK1 and JAK2 auto-phosphorylate and next phosphorylate STAT1 homodimers (27) at tyrosine 701 (Y701) reshaping them to an active parallel conformation (32, 33), that is subsequently imported in the nucleus to bind gamma activating sequences (GAS) and to drive transcription of IFN-stimulated genes (ISGs) (34–36). For a correct STAT1 activation, serine 727 (S727) also needs to be phosphorylated although the mechanism for this is less understood (37). GAS elements in promoters of ISGs can induce tetramerization of STAT1 on the DNA, through an interaction of their N-terminal domains (32, 38). In addition to homodimers, STAT1 dimerizes with other STAT proteins, such as STAT2 (27) and STAT3 (39), depending on the stimulus. IFN-α and -β trigger STAT1/2 heterodimer formation. The latter in turn forms an heterotrimeric complex by binding interferon regulatory factor 9 (IRF9) (40) that binds IFN-stimulated response elements (ISRE) in specific promoters to drive transcription (41). IL6 and IL27 trigger the formation of STAT1–STAT3 heterodimer, where STAT3 promotes overall transcription and STAT1 mostly determines the specificity (39).

In AD STAT1 GOF, increased phosphorylated STAT1 (pSTAT1) is consistently observed upon stimulation of the JAK/STAT1 pathway (using IFNγ, IFNα, or IL-27) (8). Different hypotheses have been proposed to explain the increase in pSTAT1, such as enhanced phosphorylation, impaired dephosphorylation, or improved stabilization of the active, parallel STAT1-dimer (30). However, no studies have evaluated the cellular behavior of STAT1 GOF mutants in-depth.

Most of STAT1 GOF mutations are identified in the CCD and DBD, but more recent GOF mutations are located in other STAT1 domains (2, 8). Analyzing STAT1 crystal structures (42, 43), around 70% of the GOF mutations can be mapped to the antiparallel homodimers interface (comprised of the CCD and the DBD), while the other GOF mutations are dispersed in other parts of the protein. Notably, there are only three GOF mutations described near the DNA interface (H328R, T419R, and S466R), possibly directly interacting with it.

Given the diverse functions of STAT1 and the wide range of STAT1 GOF mutations spanning all domains of the protein, together with the heterogeneous clinical presentations in STAT1 GOF (ranging from CMC to IPEX-like and CID), we hypothesized that different underlying molecular mechanisms can result in a common GOF phenotype, all leading to increased pSTAT1 upon stimulation. In this study, we employed lentiviral vector (LV) technology to generate a U3A cell model stably expressing eGFP-labeled STAT1. We selected STAT1 GOF mutants (R274W, R321S, T419R, and N574I) that are spread over the protein and associate with diverse GOF phenotypes in patients. In line with earlier reports, all GOF mutants showed higher STAT1 phosphorylation and stimulation of ISG compared to STAT1 WT. In addition, we studied the dynamic behavior of STAT1 in real-time and determined subcellular distribution, protein dynamics (nuclear import and mobility), and stoichiometry for wild-type STAT1 and the STAT1 GOF mutations located in three different domains (CCD, R274W; DBD, R321S, and T419R; LD, N574I), together with two non-clinical STAT1 controls, Y701A (non-phosphorylatable), and E411A [shown to have an enhanced affinity for DNA using electrophoretic mobility shift assay (44)].

As expected, we demonstrate comparable hyperphosphorylation and enhanced transcription of ISGs for all STAT1 GOF mutations. However, we identified three distinct STAT1 GOF phenotypes, suggesting distinct underlying mechanisms: (i) faster nuclear accumulation for R274W, (ii) reduced nuclear mobility and slower dephosphorylation for both R321S and N574I, and (iii) nuclear immobility, potentially due to enhanced binding to chromatin, for T419R.

## Materials and Methods

### Mutant Selection

The mutants R274W, R321S, and N574I were initially chosen in the context of our research hypothesis because they are present at the different domains of the STAT1 protein and because patients bearing these mutations were in follow-up at our university hospital. The T419R was additionally included as a clinical GOF mutation potentially analogous to the E411A control, since modeling predicted a similar possible direct interaction with the STAT1 target DNA.

### Cell Culture

U3A (ECACC 12021503) cells were cultured in DMEM medium (GIBCO, REF 31966-021) supplemented with 10% FCS. Cells were tested to be mycoplasma-free by PlasmoTest™, InvivoGen Europe. Cells were cultured at 37°C in a humidified atmosphere containing 5% CO2. U3A and HEK cells were cultured in DMEM (GIBCO-BRL) supplemented with 10% FBS (GIBCO, REF 10270-106), 0.01% v/v gentamicin (GIBCO-BRL). HeLa P4 cells were cultured in DMEM (GIBCO-BRL) supplemented with 10% FBS (GIBCO, REF 10270-106), 0.01% v/v gentamicin (GIBCO-BRL), 0.1% geneticin (GIBCO-BRL). HEK293T cells were obtained from ATCC (REF CRL-11268). HeLa P4 cells were a kind gift from Pierre Charneau, Institut Pasteur, Paris, France. U3A cells were purchased from Sigma, REF 12021503-1VL.

### Cloning

The pCH-EF1α-eGFP-IRES-PURO transfer plasmid was used to clone the pCH-EF1α-STAT1WT-eGFP-IRES-PURO, encoding for STAT1WT-eGFP driven by the ubiquitous promoter EF1α, followed by an Internal Ribosome Entry Site (IRES) and a resistance cassette for puromycin. This plasmid was used as a template to clone the other mutants used in this study: pCH-EF1α-STAT1Y701A-eGFP-IRES-PURO, pCH-EF1α-STAT1E411A-eGFP-IRES-PURO, pCH-EF1α-STAT1R274W-eGFP-IRES-PURO, pCH-EF1α-STAT1R321S-eGFP-IRES-PURO, pCH-EF1α-STAT1T419R-eGFP-IRES-PURO, and pCH-EF1α-STAT1N574I-eGFP-IRES-PURO. All the STAT1 fusions were tagged with an enhanced-GFP (48) (eGFP), referred in the paper as GFP.

### Lentiviral Vectors Production

Viral vectors were produced by HEK 293T cells after triple transfection with the transfer plasmid described earlier, a second-generation packaging plasmid lacking vif, vpr, vpu, and nef genes (pCMVΔR8.91) and an envelope plasmid encoding vesicular stomatitis virus G (VSV-G) protein. HEK 293T cells were seeded in 10-cm diameter cell-culture dishes at 5 × 10^6^ cells per plate in DMEM supplemented with 10% FCS. After 24 h, 20 μg of transfer plasmid, 10 μg of packaging construct, and 5 μg of envelope plasmid were diluted in 700 μl of 150 mM NaCl. 700 μl of polyethylenimine solution (PEI, Polysciences) was added slowly to the DNA mixture. DNA-PEI mix was incubated for 5 min at room temperature and then the DNA-PEI complex was added dropwise to the HEK 293T cells in DMEM supplemented with 1% FCS. The cells were incubated at 37°C in a 5% CO_2_ humidified atmosphere for 24 h, then the medium was replaced with DMEM with 10% FCS. The supernatant was harvested at day 2 and 3 post-transfection and filtered through a 0.45 μm pore-size filter (Sartorius, Minisart, Göttingen, Germany). The filtered vector particles were concentrated to 1 ml using vivaspin (Vivascience, Bornem, Belgium). Pellets were dissolved in DMEM with 10% FCS, divided in 50 μl aliquot in and stored at −80°C.

### Generation of Stable Cell Lines

U3A and HeLa cells were transduced using lentiviral vectors described earlier. Three days after transduction cells were selected with puromycin (1 mg/ml, GIBCO-BRL). The resulting cell lines were kept in culture with puromycin selection (1 mg/ml) for at least 2 weeks prior starting the experiments, to assure a stable expression of the constructs.

### Western Blot Analysis

Whole-cell lysates were obtained by lysing 2 × 10^6^ cells using 1xPBS with 1% sodium dodecyl sulfate (SDS). PE-labeled anti-STAT1 monoclonal antibody was purchased from BD bioscience (REF 612564) and used at 1:1,000 dilution. Anti STAT1α was purchased from Santa Cruz Biotechnology (REF sc-417) and used at 1:1,000 dilution. Anti GAPDH was purchased from Abcam (REF ab9485) and used at 1:2,000 dilution. All antibodies were incubated overnight at 6°C. Secondary HRP-conjugated antibody goat anti mouse (Agilent REF P0447) and goat anti rabbit (Agilent REF P0448) were used at 1:10,000 dilution and incubated for 1 h at room temperature.

### Flow Cytometry

U3A cells were stimulated with 1 U/μl of IFNγ (Roche, REF 11040596001) for 1 h. One-hour stimulation was preferred over the more common 20–30 min stimulation, because at 30 min we did not observed a complete nuclear import, while after 1 h stimulation cells showed a complete nuclear relocalization of STAT1-GFP (data not shown). Cells were trypsinized, washed twice with PBS-FBS 5% and fixed using 200 μl of Fixation Buffer (BD Biosciences, REF 554655) at room temperature for 15 min. Then they were washed again twice with PBS-FBS 5% and permeabilized on ice using pre-chilled (−20°C) Perm Buffer III (BD Biosciences, REF 558050) for 30 min. Cells were washed two more times with PBS-FBS 5% and stained using PE-labeled anti-pSTAT1 antibody (BD bioscience, REF 612564). More than 30,000 cells per sample were acquired on a BD LSRFortessa™.

### Immunohistochemistry

U3A cells were seeded at 20,000 cells/well on 8-well-chambered slide with removable wells (Ref 177445, Lab-Tek, Thermo Fisher Scientific) and incubated overnight. The cells were washed with PBS and fixed with paraformaldehyde 4% (Sigma-Aldrich) for 15 min, then washed again with PBS and stained with DAPI (Thermo Fisher Scientific). Images were acquired on a Zeiss LSM 880 at 40× magnification.

### Quantitative PCR

Quantification of IRF1, GBP1, and CXCL10 mRNA levels was performed as previously described (49). PSIP1 was used as endogenous housekeeping control. All samples were run in duplicate for 10 min at 95°C, followed by 50 cycles of 10 s at 95°C and 30 s at 55°C. Data were analyzed with iQ5 Optical System Software (BioRad, Belgium). Primers used: IRF1 FW, CCTGCCAGATATCGAGGAGG; IRF1RV, GTAGCCTGGAACTGTGTAGC; CXCL10 FW, GCAAGCCAATTTTGTCCACG; CXCL010 RV, CTTGGAAGCACTGCATCGAT; GBP1 FW, CTAGTTCTGCTGGACACCGA; GBP1 RV, CAGTTGGTCCATAGCCTGCT; PSIP1 FW, GAACTTGCTTCACTTCAGGTCACA; PSIP1 RV, TCGCCGTATTTTTTTCAGTGTAGT.

### Modeling

All crystal structures were obtained via Protein Data Bank (rcsb.org): unphosphorylated, antiparallel STAT1 (reference 1YVL) and DNA-bound parallel STAT1 (reference 1BF5). PyMOL software was used to analyze the relative position of the different STAT1 mutants and their distance from DNA.

### Nuclear Import Time Lapse

HeLa P4 cells stably expressing WT STAT1-GFP and STAT1-GOF-GFP mutants were seeded at 40,000 cells/well in a 96-well-plate and grown overnight. The cells were then washed and Hoechst 33342 (Thermo Fisher Scientific) was added to the medium to a final concentration of 0.1 μg/ml for 5 min. Then, the cells were washed with 1xPBS and the medium replaced with DMEM and they were then incubated in an Arrayscan XTITM (Thermo Fisher Scientific) for 1 h, before being stimulated with 1 U/μl IFNγ. For live-cell imaging, images were acquired every 2 min for a total of 1 h (25–30 images/h). The software ImageJ-FIJI was used to analyze the images using an in-house macro. Briefly, a mask was created on the Hoechst channel, after removing the background and enhancing the local contrast. Nuclear signal was measured on the GFP channel and the ratio between nuclear and total signal was recorded. Data were plotted using Graphpad Prism 8.0 and non-linear regression curves were calculated, using the built in One-phase association equation: *Y* = *Y*0 + [plateau –*Y*0) × (1 – exp(–*K* × *X*)]. Where *Y*0 is the *Y* value at time 0, *K* is the rate constant expressed in min^−1^ and plateau is the *Y* value at infinite time.

### STAT1 Dephosphorylation Assay

Five hundred thousand STAT1-complemented U3A cells, together with untransduced control U3A cells were seeded in 6 well-plates. Twenty-four hours later, cells were stimulated with IFNγ (1 U/μl for 1 h) and then ruxolitinib (10 μM final concentration, Selleckchem, REFS 1378) was added to the medium, to stop further phosphorylation. The cells were then incubated and lysed at fixed time points indicated in the figures. Phosphorylation level of STAT1 was measured via Western blot using anti-pSTAT antibody (BD bioscience, REF 612564).

### Raster Image Correlation Spectroscopy (RICS)

Before imaging, the respective stable STAT1 expressing U3A cells were seeded at 20,000 cells/well on 8-well-chambered Coverglass with a No. 1 borosilicate glass bottom (REF 155411, Lab-Tek, Thermo Fisher Scientific) and incubated overnight. The cells were kept at 37°C and were either stimulated with IFNγ (final concentration 1 U/μl for 1 h) or left unstimulated. For each RICS measurement, 200 frames were acquired on a Zeiss LSM880 confocal laser-scanning microscope using a Plan-Apochromat 63x/1.4 Oil DIC M27 objective. Excitation occurred using a 488 nm Ar-ion laser (1 μW in the sample) and the emitted light was detected between 489 and 695 nm using the Zeiss Quasar GaAsP detector operated in photon counting mode. Images were acquired through cell nucleus and cytosol at 3–5 μm above the coverslip and contain 256 × 256 pixels^2^ at a pixel size of 50 nm (image size 12.85 × 12.85 μm^2^). Pixel dwell, line, and image times were 8.19 μs, 4.92 ms and 1.26 s, respectively. The images were analyzed using the software package PAM (pulsed interleaved excitation analysis with MATLAB, The MathWorks, Natwick, MA). The software and manual about the software PAM, can be found at https://pam.readthedocs.io/en/latest/ (45). Briefly, either the cell cytosol or nucleus are selected by intensity thresholding. After a moving average preprocessing, the mean spatial autocorrelation function is calculated using the ARICS algorithm (46). To obtain quantitative values of diffusion coefficient, D, and average number of molecules in the focus, N, a 3D Gaussian model was used for fitting the autocorrelation function based on Digman et al. (47). In addition, the molecular brightness ε was obtained by dividing the mean intensity in the observation volume F by the number of molecules in the observation volume.

Plasmids encoding monomeric eGPF, dimeric eGFP-eGFP fusion, and tetrameric eGFP-eGFP-eGFP-eGFP fusion were a kind gift of Prof. Masataka Kinjo and Prof. Shintaro Mikuni [Laboratory of Molecular Cell Dynamics, Faculty of Advanced Life Science, Hokkaido University, Sapporo, Japan (48)].

### Statistical Analysis

Data were represented as means ± standard deviation. Statistical analysis was performed using Graphpad Prism 8.0. Student's *t*-test or One-way ANOVA (with Dunnet's or Tukey's *post-hoc* test) were used as mentioned for each experiment. Statistical significance was represented with asterisks: ^*^p < 0.05, ^**^p < 0.01, ^***^p < 0.001, ^****^p < 0.0001.

### Biosafety

Lentiviral vectors used have a self-inactivating (SIN) design and were produced in a BSL-2+ laboratory (49). Transduced cells were cultured in a BSL-2+ lab until the supernatant showed to be p24 ELISA negative (no residual LV or replication competent lentiviruses formed; Alliance HIV-1 p24 ELISA kit: Perkin Elmer), ~3 weeks after transduction, before being used for experiments. All experiments not requiring a confocal microscope were performed in a BSL-2+ lab.

## Results

### Generation of Stable STAT1-GFP Expressing Cell Lines

Constructs for the different STAT1 controls and GOF mutants were designed and cloned to carry a C-terminal GFP tag in vector transfer plasmids for lentiviral vector production. The resulting lentiviral vectors were used to generate stable cell lines. Following transduction, U3A (a STAT1^−/−^) cell line stably expressing STAT1-GFP wild type (referred to as STAT1 WT) was selected with puromycin. Next to STAT1 WT, we generated cell lines for a selection of STAT1 GOF mutants that are spread over the protein and associate with diverse GOF phenotypes in patients (hereafter referred to as R274W, R321S, and N574I) and two STAT1 control constructs (hereafter referred to as E411A, Y701A; Figure 1). Y701A cannot be phosphorylated and serves as a negative control in the phosphorylation assays and ISG expression assays. E411A is an artificial STAT1 mutation that we included as a positive control, since it has displayed enhanced affinity to DNA in an EMSA assay (44).

Correct expression of the respective STAT1-GFP proteins was corroborated via Western blot analysis (for all constructs this was at the expected size for STAT1-GFP, 123 kDa Supplementary Figure 1).

#### Stable STAT1-GFP-GOF U3A Cell Lines Show Enhanced STAT1 Phosphorylation and Transcription Factor Activity

U3A cells (non-transduced (NT), transduced with STAT1-GFP WT or the respective GOF mutants) were stimulated with IFNγ for 1 h followed by collection of whole cell lysate. STAT1 phosphorylation was measured by Western blot using pSTAT1 specific antibody. No pSTAT1 was detected upon IFNγ stimulation in non-transduced cells (NT) or the Y701A expressing cells, in contrast to U3A cells stably expressing STAT1 WT or GOF mutants (Figure 1B). Quantification of the respective pSTAT1 bands for different experiments (*n* = 5) demonstrated a 2-fold or more increase of pSTAT1 signal for the STAT1 GOF mutants (R274W, R321S, N574I) compared to STAT1 WT (Figure 1C). In line, the E411A control resulted in 3-fold increased phosphorylation. Of note, the phosphorylation level of R274W was consistently lower than that of R321S in Western blot analysis (*p* = 0.0089, one-way ANOVA with Dunnett's correction) and N574I (albeit not reaching statistical significance, *p* = 0.20, One-way ANOVA with Dunnett's correction).

In parallel, we assessed STAT1 phosphorylation before and after IFNγ stimulation using flow cytometry analysis. In resting conditions pSTAT1 signal was equivalent for all cell lines and in line with NT U3A cells (Figure 1D), while 20 min following stimulation with IFNγ, STAT1 WT, and GOF mutants showed a pronounced increase in phosphorylation compared with NT cells and Y701A cells (Figure 1E). Quantification demonstrated a 3.6-fold increase in phosphorylation [pSTAT1 mean fluorescence intensity (MFI)] upon IFNγ stimulation for STAT1 WT complemented cells relative to Y701A, and an additional 1.5- to 2-fold increase for the different GOF mutants compared with STAT1 WT (Figures 1F,G, prior and following IFNγ stimulation, respectively), in line with a STAT1 GOF phenotype and data obtained from patient-derived PBMCs (20). The E411A control was also hyperphosphorylated in range with the clinically observed GOF mutants in FACS analysis (Figure 1G), although this did not reach statistical significance compared with STAT1 WT (*p* = 0.063).

#### Nuclear Translocation Is Not Affected for STAT1 GOF Mutants

**Figure 2:**
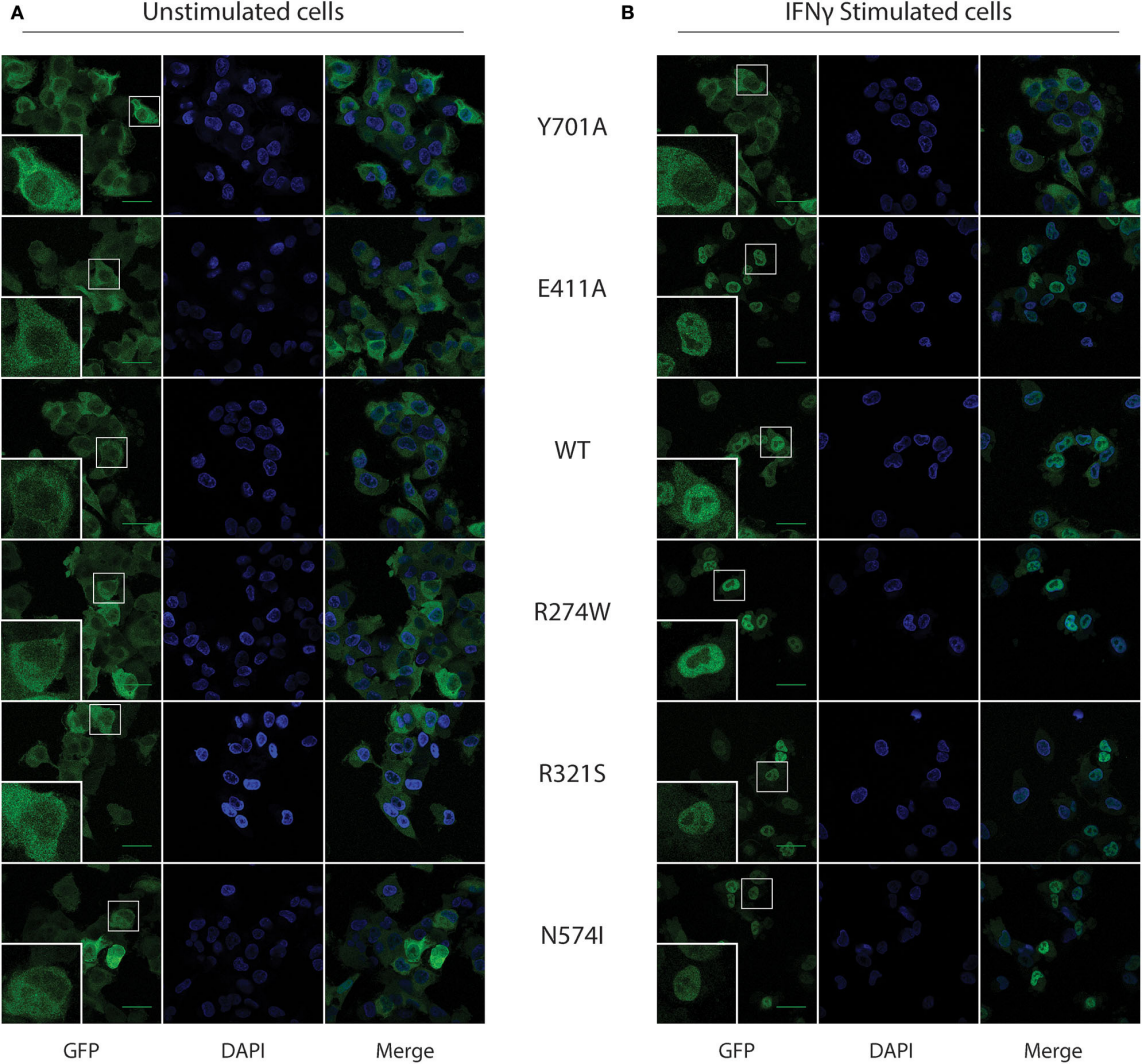
STAT1-GFP subcellular localization in U3A cells before and after IFNγ stimulus. **(A)** Confocal images for the respective STAT1 GFP expressing, unstimulated, U3A cells. **(B)** U3A cells after 1 h of IFNγ (1 U/μl) stimulation. Green scale bar is 40 μm. Bottom left inset in each image shows a zoom-in of the cell in the smaller white box.

In resting conditions, STAT1 is known to mainly reside in the cytoplasm, while upon IFNγ stimulation it translocates to the nucleus, achieving a complete nuclear import 1 h after IFNγ stimulation. We corroborated STAT1-GFP subcellular localization in our U3A cell model using confocal microscopy in resting and IFNγ-stimulated conditions (Figures 2A,B, respectively). As expected, the Y701A, unable to be phosphorylated, did not translocate to the nucleus upon stimulation (Figure 2). Conversely, STAT1 WT and all the GOF mutants almost completely relocated to the nucleus 1 h after IFNγ stimulation.

#### All STAT1 GOF Mutants Result in Higher Expression Levels of ISGs Than STAT1 WT Upon Stimulation

**Figure 3:**
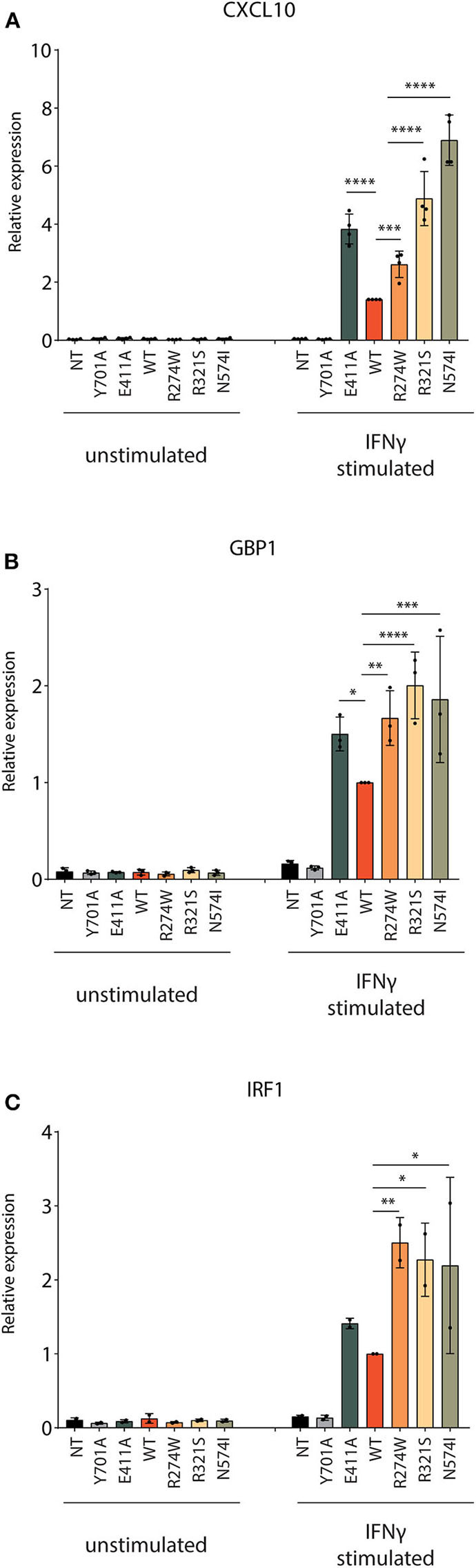
Quantification of ISGs expression level. The mRNA expression level of three ISGs **(A)** CXCL10, **(B)** GBP1 and **(C)** IRF1 was measured before and after 4 h stimulation with IFNγ (1 U/μl). Expression levels were normalized for housekeeping *PSIP1* mRNA expression and plotted as fold increase compared to IFNγ stimulated STAT1 WT condition for each experiment. The experiment was independently repeated 2–4 times for each gene and pooled data are presented. Data are represented in bar graphs as mean ± standard deviation. Statistical analysis: One-way ANOVA with Tukey's *post-hoc* test. *P < 0.05; **P < 0.01; ***P < 0.001; ****P < 0.0001.

Next, the responsiveness to IFNγ for the respective cell lines was measured by qPCR for three ISGs: CXCL10, GBP1, and IRF1. Four hours after IFNγ stimulation, STAT1 WT, STAT1 GOF mutants, and E411A, but not Y701A and the NT U3A cells, showed a significantly increased ISG mRNA expression (Figures 3A–C). After IFNγ stimulation, all GOF mutants showed expression levels higher than STAT1 WT for the chosen ISGs. A 2- to 7-fold increase compared to STAT1 WT was observed after IFNγ stimulation for the STAT1 GOF mutants for all the ISGs analyzed, in line with the GOF phenotype. Whereas GBP1 and IRF1 were stimulated to the same extent by R274W, R321S, N574I, there was a substantial difference for these three mutants when analyzing CXCL10 expression (Figure 3A). The R274W mutant consistently showed an intermediate phenotype for CXCL10 expression in between STAT1 WT and the R321S and N571I mutants, in line with the intermediate hyperphosphorylation measured via Western blot (Figures 1B,C).

Together, these experiments show that the respective STAT1-GFP constructs in U3A cells are stably expressed and result in functional STAT1-GFP proteins (phosphorylation and nuclear translocation) that allow stimulation of ISGs.

### STAT1 GOF Mutants Exhibit Distinct Molecular Behaviors in the Nucleus Upon IFNγ Stimulation

Measurement of pSTAT1 and expression level of ISGs only provide a static view on what happens after IFNγ stimulation, and displays the same phenotype for all STAT1 GOF mutations, in line with published reports. In order to better understand the STAT1 phosphorylation/dephosphorylation cycle, we assessed STAT1 nuclear import by using live-cell-imaging time lapse to determine nuclear accumulation rate constants. Further, we evaluated STAT1 cytoplasmic and nuclear molecular dynamics using Raster Imaging Correlation Spectroscopy (RICS) analysis and we measured the respective dephosphorylation rates of STAT1 WT and GOF mutants.

#### R274W STAT1 GOF Mutant Accumulates Faster in the Nucleus

Nuclear accumulation of STAT1 WT and the respective STAT1 mutants was measured using an ArrayScan XTI^TM^, imaging for 1 h every 2 min after stimulation with IFNγ. We were unable to perform this experiment in STAT1 complemented U3A cells due to phototoxicity, hence we generated stable STAT1-GFP expressing HeLa cell lines. The respective STAT1 constructs showed equal protein expression, hyperphosphorylation and nuclear relocalization in response to IFNγ stimulus, in line with the results obtained with the U3A cell lines (Supplementary Figures 2A–C).

**Figure 4:**
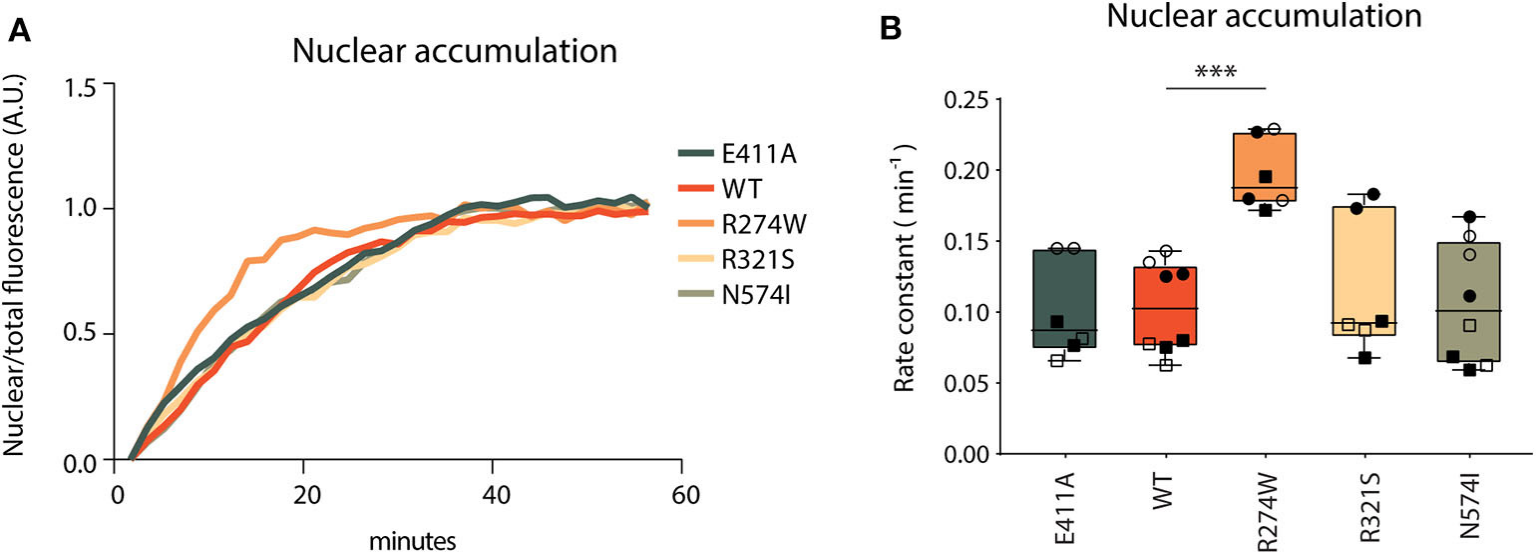
Nuclear accumulation. **(A)** Nuclear accumulation of STAT1-GFP signal over a 60 min time-lapse in stable STAT1 expressing HeLa cells, each sample was normalized on its last data point. Representative image of non-normalized data is reported in Supplementary Figure 6. Since the Y701A control cannot be phosphorylated, the lack of nuclear accumulation is in line with expectations and no curve fitting was possible for this mutant (Supplementary Figure 6; dotted gray line). **(B)** Nuclear accumulation rate constants, calculated as described in section Methods. The experiment was repeated at least two times for each STAT1 cell line and each biological repeat is represented by a different symbol. Statistical analysis was performed by comparing each other sample with STAT1 WT using One-Way ANOVA with Dunnett's *post-hoc* test. Data are represented in box and whiskers plot, with all data points shown. ***P < 0.001.

Nuclear accumulation was calculated as the ratio of nuclear fluorescent signal over the total fluorescent signal of the image to correct for photobleaching, and plotted over time. Non-linear regression analysis was used to generate nuclear accumulation curves and calculate rate constants (Figure 4A). E411A control, together with R321S and N574I did not show significant differences in nuclear accumulation relative to STAT1 WT (Figure 4B). Conversely, R274W accumulated significantly faster in the nucleus upon IFNγ stimulation with a rate constant being double that of WT (0.2 ± 0.02 min^−1^ and 0.1 ± 0.03 min^−1^, respectively). This, together with the position of the R274W mutation at the interface of the antiparallel homodimer of inactive STAT1 (43), suggests a destabilization of the inactive antiparallel conformation, leading to a faster transition to the parallel conformation in response to the IFNγ stimulus.

#### E411A, R321S, and N571I Show a Severely Affected Nuclear Mobility

We next used RICS to assess the molecule brightness and diffusion constant in our STAT1 back-complemented U3A cell models. Molecule brightness relates to the STAT1 oligomerization status or stoichiometry, where brighter molecules represent higher order homo-oligomers, such as dimer and tetramers. The diffusion constant is a direct marker for binding to other proteins/complexes or immobile fractions: the larger the interactor, the more reduced the mobility of STAT1 GFP will be. As a control for our RICS settings, we transiently transfected a plasmid expressing a GFP monomer, a GFP-GFP fusion (GFP dimer), and a GFP-GFP-GFP-GFP fusion (GFP tetramer) and analyzed by RICS (Supplementary Figure 3). RICS showed an increase of brightness for GFP dimers (17.2 ± 0.5 kHz) and GFP tetramers (24.4 ± 5.9 kHz) relative to GFP monomers (9.5 ± 1.1 kHz), confirming the correlation between brightness and stoichiometry. This was accompanied by a gradual decrease in mobility (15.8 ± 1.2 μm^2^/s, 12.0 ± 0.32 μm^2^/s, and 7.2 ± 1.52 μm^2^/s diffusion constants for GFP monomers, dimers and tetramers, respectively) confirming that higher-molecular-weight molecules exhibit a lower mobility and/or sense a higher local viscosity (Supplementary Figure 3).

**Figure 5:**
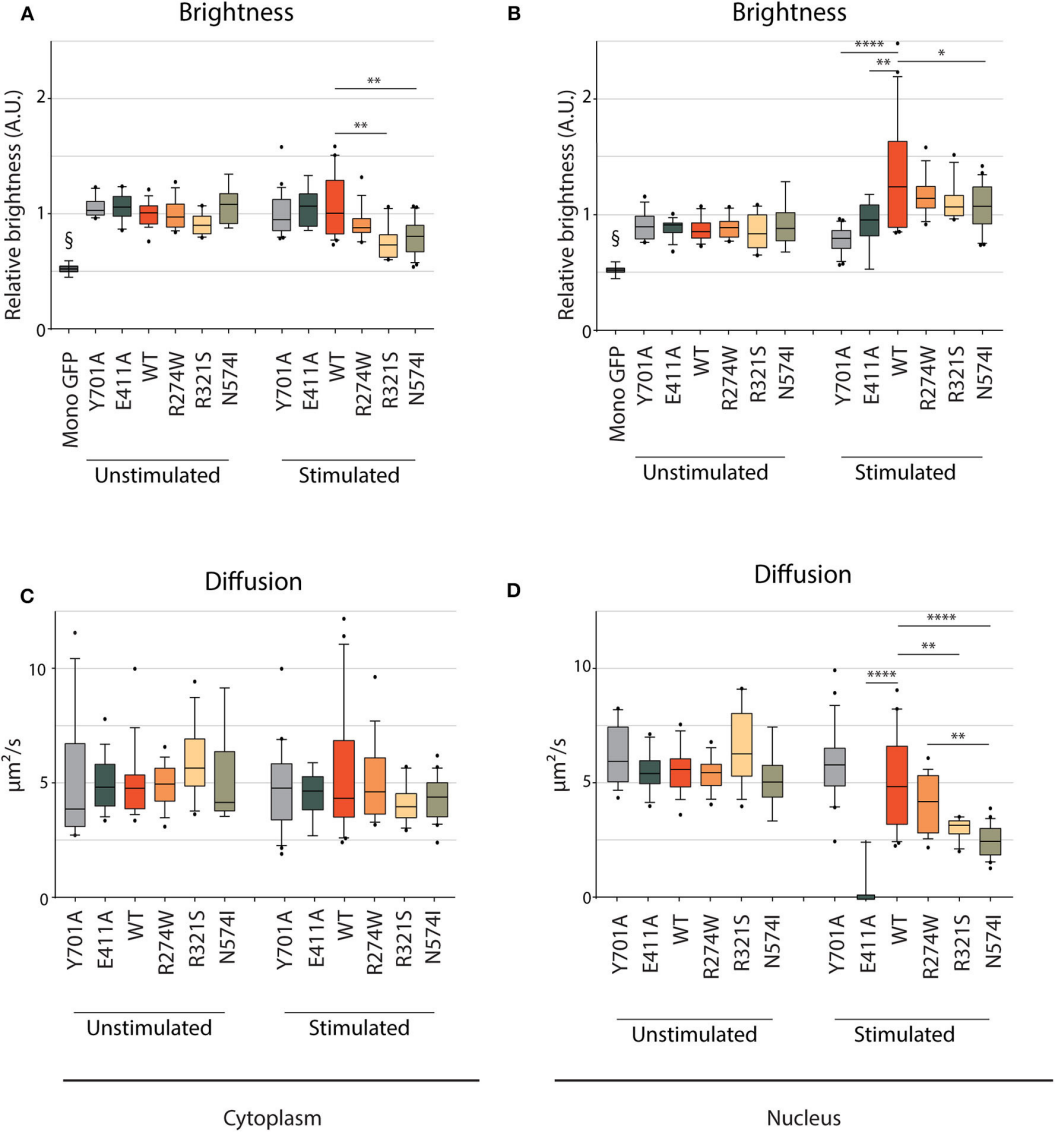
RICS analysis of STAT1-GFP WT and GOF mutations in U3A cells. Brightness **(A,B)** and average diffusion constant as a marker of mobility **(C,D)** measurement of cytoplasmic and nuclear STAT1-GFP fusions in unstimulated U3A cells and cells stimulated with IFNγ (1 U/μl). §GFP monomers, measured in HEK cells. Brightness is expressed in arbitrary units and plotted as fold increase compared to cytoplasmic WT. Data in panels **(A–D)** (*n* > 7) are represented in box and whiskers plots. The central line represents the median, while the box extends from the 25th to 75th percentiles with whiskers spanning 10–90% intervals. Dots outside these intervals are represented as single dots. Statistical analysis: One-way ANOVA with Tukey's *post-hoc* test. *P < 0.05; **P < 0.01; ****P < 0.0001.

We studied STAT1 WT and GOF nuclear/cytoplasmic dynamics in unstimulated conditions and after IFNγ stimulation, to assess the behavior of the different GOF mutants in each condition (Supplementary Figure 4). A representative image frame of unstimulated and IFNγ stimulated cells used for RICS analysis is shown in Supplementary Figures 5A,B. In unstimulated conditions, the brightness was similar for STAT1 WT and the different GOF mutants in both the cytoplasm and the nucleus (Figures 5A,B, respectively; Supplementary Figure 5C). Comparing brightness of the STAT1 proteins to the brightness of monomeric GFP shows that STAT1 mainly resides as a homodimer in the cytoplasm. Following IFNγ stimulation, brightness of R274W, E411A and Y701A remained the same as STAT1 WT in the cytoplasm (Figure 5A). Conversely, R321S and N574I mutants showed a small significant drop in relative brightness compared to STAT1 WT (0.69 ± 0.14 A.U. and 0.74 ± 0.15 A.U. compared to 1 ± 0.27 A.U.) indicating that R321S and N574I exhibit a lower affinity for homodimerization in the cytoplasm upon IFNγ stimulation. The fact that the mobility in the cytoplasm under these conditions was in line with that of STAT1 WT, suggests concomitant heterodimerization of these mutants with a protein comparable in size to STAT1.

After IFNγ stimulation, no significant change was observed in brightness for E411A and Y701A controls compared to the unstimulated condition in the nuclear compartment, indicating that both are present as homodimers. Conversely, STAT1 WT brightness significantly increased in the nucleus compared to the cytoplasm (1.32 ± 0.46 A.U. and 1 ± 0.27 A.U., respectively (*p* = 0.0005, one-way ANOVA with Tukey's *post-hoc* test, Supplementary Figures 4A,B, 5D), proving almost complete tetramerization in the nucleus upon IFNγ stimulation. When comparing with STAT1 WT, similar results were obtained in the nucleus for all GOF mutations tested: R274W, R321S, and N574I showed a significant increase of nuclear brightness when compared to cytoplasmic STAT1 WT [17, 11, and 7%, respectively (the nuclear brightness is significantly lower compared to STAT1 WT only for N574I, *p* = 0.019], suggesting that the oligomerization of STAT1 GOF mutants is comparable to STAT1 WT. When comparing the effect of IFNγ stimulation for each mutant, only E411A did not show a significant increase in brightness in the nucleus compared to Y701A, while STAT1 WT, R274W, R321S, and to a lesser extent, N574I demonstrated a significant increase suggesting at least partial tetramerization.

In parallel, the diffusion constants were determined, demonstrating that mobility in the cytoplasm was similar for STAT1 WT and the different STAT1 GOF mutants in both unstimulated and stimulated conditions (Figure 5C). In the nucleus (Figure 5D), the mobility remained unchanged in unstimulated condition for all the tested samples. Following IFNγ stimulation, comparable diffusion constants were measured in the nucleus for STAT1 WT, R274W mutant, and Y701A control (4.9 ± 2.0 μm^2^/s, 4.1 ± 1.2 μm^2^/s, and 5.8 ± 1.6 μm^2^/s, respectively). Interestingly, R321S and N574I GOF mutants diffused significantly slower than STAT1 WT in these conditions (3.0 ± 0.43 μm^2^/s and 2.4 ± 0.67 μm^2^/s, respectively). Moreover, the diffusion constant for E411A was reduced to 0.3 ± 0.8 μm^2^/s (compared to 4.9 ± 2.0 μm^2^/s for STAT1 WT), indicating near nuclear immobility. The E411A mutation is predicted to have enhanced affinity to GAS sites (44) and therefore was included as a control in our experiments, but this mutation has not been reported in patients. The near immobility of E411A may be explained by an increased affinity for an immobile fraction in the nucleus, such as chromatin. The fact that the mobility of R321S and N574I was significantly reduced in the nucleus compared to STAT1 WT, suggests that these GOF mutants form a higher order complex that is less mobile or that they have higher affinity for immobile fractions in the nucleus.

Together these results allowed us to discern R274W as a GOF mutant that has the same mobility and oligomerizes as STAT1 WT, whereas R321S and N574I have a significantly lower nuclear mobility relative to STAT1 WT.

#### R321S, N574I, and E411A Dephosphorylate Slower Than WT and R274W STAT1

**Figure 6:**
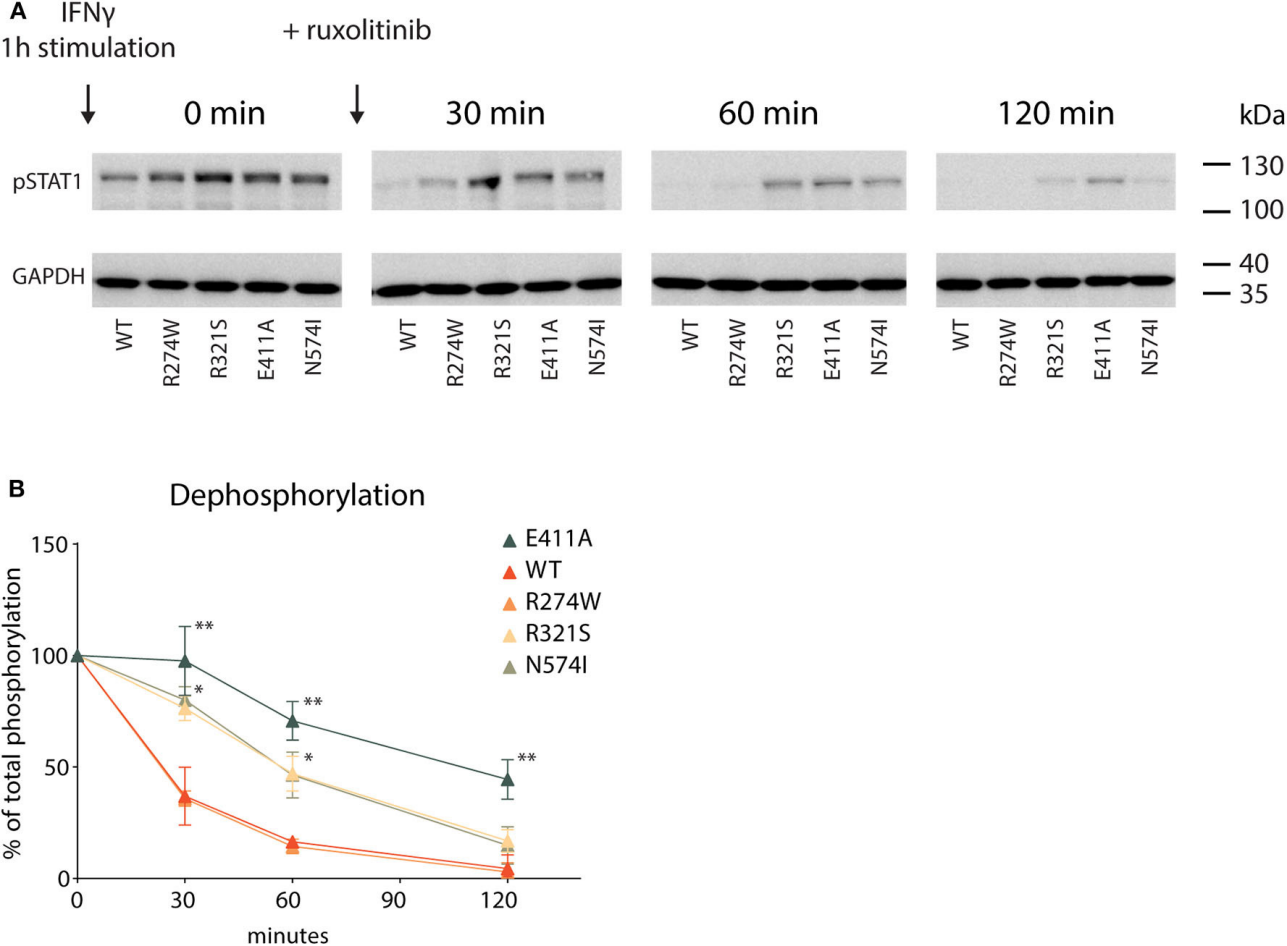
Dephosphorylation kinetic **(A)** Representative Western blot of U3A cell lysates. Cells were first treated with IFNγ (1 U/μl) for 1 h (minute 0) and then DMEM medium supplemented with ruxolitinib (10 μM) was added. Whole cell lysate was collected 0, 30, 60, or 120 min after ruxolitinib addition. **(B)** Quantification of pSTAT1 levels, plotted as % of maximum phosphorylation measured at minute 0. Statistical analysis: Student's *t*-test, comparing every time point to the corresponding WT. *P < 0.05; **P < 0.01.

Lastly, following the activation cycle of STAT1, we measured STAT1 dephosphorylation for STAT1 WT and GOF mutants. The respective U3A cell lines were stimulated for 1 h with IFNγ, after which further phosphorylation was halted by addition of the JAK inhibitor ruxolitinib. Subsequently, dephosphorylation was measured at specific time points following addition of ruxolitinib (0–30–60–120 min, Figure 6A). IFNγ stimulation resulted in hyperphosphorylation for each of the GOF mutants compared to STAT1 WT (Figure 6A, time point 0), corroborating earlier data (Figures 1A,B). To determine the dephosphorylation rate, pSTAT1 bands were scanned and plotted over time as the percentage relative to maximum phosphorylation (time point 0) for each STAT1 construct (Figure 6B). Three distinct kinetics of dephosphorylation could be observed: (i) STAT1 WT and R274W were indistinguishable and dephosphorylated the fastest; (ii) R321S and N574I shared the same dephosphorylation kinetic, significantly slower than WT; and (iii) the E411A control displayed the slowest dephosphorylation.

#### T419R GOF Mutant Becomes Near Immobile Upon IFNγ Stimulation

**Figure 7:**
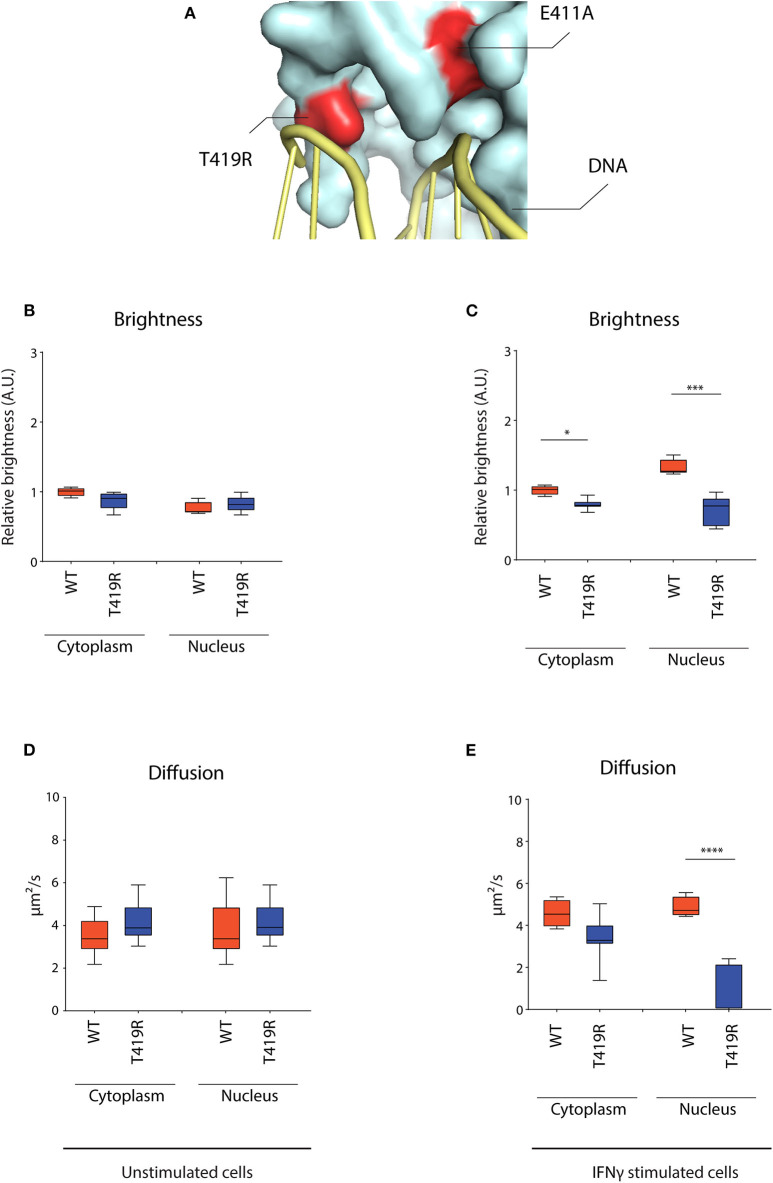
RICS analysis of STAT1-GFP WT and T419R GOF mutant in U3A cells. **(A)** Detail of crystal structure of STAT1 bound to DNA. Highlighted in red are the T419R and the E411A mutations, situated in close proximity of the DNA. **(B)** Average brightness in unstimulated condition and **(C)** after IFNγ stimulation. **(D)** Average diffusion constant in unstimulated condition and **(E)** after IFNγ stimulation. Data in panels **(B–E)** (*n* > 7) are represented in box and whiskers plot. The central line represents the median, while the box extends from the 25th to 75th percentiles with whiskers spanning 10–90% intervals. Dots outside these intervals are represented as single dots. Statistical analysis: One-way ANOVA with Tukey's *post-hoc* test. *P < 0.05; ***P < 0.001; ****P < 0.0001.

After observing the markedly lowered mobility for the E411A control, we wondered whether STAT1 GOF mutants described in patients might share a similar molecular mechanism. Combining the crystal structure data of the STAT1 dimer bound to DNA (42) and the currently described STAT1 GOF mutations, allowed us to identify the T419R GOF mutation (8). Both E411 and T419 amino acids locate in close proximity of the DNA and their respective mutations (E411A and T419R) result in similar charge difference (+1) (Figure 7A). We therefore hypothesized T419R to closely resemble the E411A phenotype.

RICS analysis for T419R demonstrated a similar nuclear dynamic behavior as described earlier for E411A, whereas not being significantly different from STAT1 WT in unstimulated condition (Figures 7B,D) and near immobilized in the nucleus after IFNγ stimulation (Figure 7E). T419R also showed no increase in brightness in the nucleus compared to the cytoplasm after IFNγ stimulation (which was observed for STAT1 WT and the other STAT1 GOF) (Figure 7C), suggesting absence of homotetramer formation. These findings validate our analysis method, and suggest a distinct molecular mechanism for the T419R GOF mutant (and perhaps other GOF mutants interacting with the DNA such as H328R and S466R) compared with the other STAT1 GOF mutants already described.

## Discussion

We demonstrated that different mutations, associated with STAT1 GOF, result in a STAT1 GOF phenotype via distinct mechanisms using real-time imaging and tracking of GFP-tagged proteins. Our results (summarized in Table 1) are in line with previous reports (50–52), indicating that different molecular mechanisms can result in a STAT1 GOF phenotype. It remains unknown whether these differences (altered nuclear mobility, increased DNA binding, or increased nuclear import) are biologically relevant in influencing the patients' phenotype. However, STAT1 GOF presents with an unexplained heterogeneity in clinical phenotypes, all with an underlying increased phosphorylation of STAT1. While all STAT1 GOF result in common changes responsible for the typical STAT1 GOF phenotype dominated by CMC, and respiratory infections, and auto-immunity to a lesser extent, we speculate that subtle disease-mechanism specific differences might influence tendency for more diverse phenotypes such as IPEX-like and CID without CMC. In that respect the study of T385M (associating with autoimmune manifestations) and C324F and I294T (associating with development of combined immunodeficiency) would be instrumental. The responsiveness to small molecules such as ruxolitinib has been reported to be variable (13, 18, 20, 21, 53). Thus, far two patients bearing the R274W mutation have been reported to have received ruxolitinib (20, 53). Zimmerman et al. (20) reported one patient that was treated with ruxolitinib for 4 weeks, but the patient's condition did not improve and the treatment was suspended. Vargas-Hernandez et al. (53) described three more patients bearing the R274W mutation of whom one was treated with ruxolitinib for 2 weeks, but failed to rescue NK cell cytotoxicity, possibly due to the short duration of the treatment. Interestingly, the conditions of one patient with the R274Q mutation, similar to the R274W described in our work, was reported in 2016 (21, 54) to have improved after starting a ruxolitinib treatment.

**Table 1:** Schematic summary of the molecular phenotypes observed for the different STAT1 mutants and controls analyzed in this study.

	**Phosphorylation level**	**ISGs expression**	**Nuclear accumulation rate**	**Oligomerization**	**Nuclear mobility**	**Dephosphorylation**
Y701A	0	0	NA	**Dimers**	Like control	NA
E411A	↑*↑↑*	↑*↑↑*	Like control	**Dimers**	**Near immobile**	**Slowest**
STAT1 WT	Control (↑)	Control (↑)	Control	Control (dimers and tetramers)	Control	Control
R274W	↑↑	↑↑	**Increased**	Like control	Like control	Like control
R321S	↑*↑↑*	↑*↑↑*	Like control	Like control	**Slower**	**Slower** (Intermediate)
N574I	↑*↑↑*	↑*↑↑*	Like control	**Less tetramerization than control (dimers and tetramers)**	**Slower**	**Slower** (Intermediate)
T419R				**Dimers**	**Near immobile**	

*In bold, differences compared to STAT1 WT*.

In this study, we use real time measurement of STAT1-GFP mobility, stoichiometry and nuclear import with a precision level novel to the field. Next, our work allowed us to disentangle differences in subcellular behavior for different STAT1 GOF mutations.

Firstly, we studied STAT1 R274W, one of the most common STAT1 GOF mutations (accounting for 12% of the patients reported). The R274W mutation has been studied earlier in detail by Petersen et al. (54). Here, JAK-mediated phosphorylation rate as well as Tc45-mediated dephosphorylation were unaltered compared with wild type, yet a premature nuclear accumulation upon IFNγ stimulation was observed, in line with our results. On the other hand, Fujiki et al. (50) studied the R274Q mutation, reporting an impaired dephosphorylation for this mutant. Finally, Zimmerman et al. (52) included monocytes from 14 STAT1 GOF patients, of whom 1 bearing the R274W and 2 the R274Q mutant. They observed a similar dephosphorylation rate for the pooled GOF mutants compared with the healthy controls. While all groups showed hyperphosphorylation upon IFNγ for the different GOF mutants, no differences were observed when dephosphorylation rate was expressed as a decay relative to the peak pSTAT1 level for R274W in our study, and in line with the results from Petersen et al. (54), or for the pooled GOF mutants in Zimmerman et al. (52).

We also demonstrated a comparable nuclear mobility and stoichiometry for R274W and STAT1 WT, but an increased nuclear accumulation compared to STAT1 WT and that of the other studied GOF mutants. Experiments were performed in the established STAT1^−/−^ U3A cell model, except for nuclear accumulation rate, which was assessed in HeLa cells that are less susceptible to photo toxicity than U3A. In addition, HeLa cells express endogenous STAT1, thereby more closely resembling the heterozygous situation of patient cells. Using this cell-model we observed a comparable import rate for STAT1 GOF in line with STAT1 WT, except for the R274W mutant that was imported twice as fast. Interestingly, Petersen et al. (54) also demonstrated a faster nuclear import for the R274W mutant. This, combined with the position of the R274W mutation (at the interface of antiparallel homodimer) may suggest that the antiparallel inactive conformation of STAT1 is destabilized, which in turn may result in a faster phosphorylation or transition to the phosphorylated homodimeric state and nuclear accumulation, while leaving nuclear mobility, stoichiometry, and dephosphorylation unaffected.

The second molecular mechanism that we described is the one of the E411A control and T419R GOF mutant. Analysis of STAT1 WT crystal structure bound to DNA (42), shows that E411 and T419 residues are in close proximity to genomic DNA. Mutation E->A and T->R could enhance the affinity for the negatively charged genomic DNA. In line, for both E411A and T419R, we observed a 95 and 86% drop in nuclear diffusion constant, respectively, upon IFNγ stimulation. Moreover, brightness measurements suggest a lack of tetramerization on ISGs promoters for these mutants. In this case, the GOF phenotype could be explained by an enhanced transcription of ISGs, as well as by a steric interference of this STAT1 GOF on the promoters of other, for instance STAT3, stimulated genes. Other STAT1 GOF mutations, in close proximity to the DNA (such as H328R and S466R) might be associated with this same molecular mechanism. Importantly, E411A has not been described in patients, although, using our cell model, its hyperphosphorylation and enhanced transcription of ISGs mark it as a possible STAT1 GOF mutation. The increased expression of ISGs after IFNγ stimulation is partially in contrast with the previous finding from Koch et al. (44), where the activity of the E411A mutant was tested with a luciferase assay and proved to be reduced compared to STAT1 WT. This discrepancy might be due to the measurement methods, were we measured the expression of endogenous ISGs in a stably expressing STAT1 E411A cell line and Koch et al. (44) measured STAT1 E411A activity on a GAS-luciferase construct following transient transfection. The direct correlation between delayed dephosphorylation kinetics and drop of nuclear mobility after IFNγ stimulation, suggests that the increased affinity for chromatin stabilizes the STAT1-chromatin complex, and thereby prevents it from being dephosphorylated.

Finally, we identified a third molecular mechanism for the R321S and the N574I STAT1 GOF mutants, that showed decreased nuclear mobility, associated with a slower dephosphorylation, whereas nuclear accumulation speed was in line with STAT1 WT. Together these data are not sufficient to pinpoint a specific molecular mechanism since slower dephosphorylation might be caused by an enhanced DNA- or chromatin binding, or vice versa. Recently, Zimmerman et al. (52) evaluated PBMCs and monocytes of 14 STAT1 GOF patients with 10 different mutations (including R274W, *n* = 1, and R321S, *n* = 3, also present in our study) using flow cytometry. A similar dephosphorylation rate was observed in CD14^+^ monocytes by pooling several mutations together, in line with our results for the R274W mutant. For the R321S mutant, we observed both a slower dephosphorylation and a reduced nuclear mobility, while Zimmerman et al. did not observe a reduction in dephosphorylation rate. This discrepancy might be explained by differences in cell type, GFP-tagging or the fact that different mutations were compiled rather than individually studied. However, it is important to notice that, in our study, the dephosphorylation of the STAT1 GOF correlates with the mobility in the nucleus. For example, the R274W mutant has the same mobility in the nucleus and comparable dephosphorylation rate as WT, while the E411A mutant is almost immobile in the nucleus and has the slowest dephosphorylation rate. Zimmerman et al. (52) hypothesized that the observed increased pSTAT1 levels in STAT1 GOF is the result of more STAT1 protein being present in the cells. Here, we demonstrate that an increase in pSTAT1 (Figure 1) and STAT1 activity (Figure 3) can occur even with comparable expression levels of STAT1 WT and the different STAT1 GOF mutants (Supplementary Figure 1). This suggests that the increased amount of STAT1 protein in STAT1 GOF is caused by the positive feedback of STAT1 on its own transcription and is not the cause of the increased phosphorylation or activity as transcription factor (55). Primary cells as used by Zimmerman et al. are most relevant to study STAT1 GOF mutants. However, the techniques used to monitor intracellular STAT1 behavior in real time require a fluorescent tag and near-immobile cells, thereby limiting these analyses to adherent cell lines. A next step would be to study this behavior in primary adherent cells where endogenous STAT1 is tagged with a fluorophore.

The exact mechanism remains to be elucidated for the N571I GOF mutant which is present in the linker domain and far from the homodimer interface and from the DNA, based on the available crystal structures of STAT1 (42, 43). Additional DNA binding assays might provide valuable information. Interestingly, recently Zuo et al. (56) reported on the linear ubiquitination of K511 and K652 in STAT1 that inhibit STAT1 from binding to the Type-I interferon receptor IFNAR2 and contributes to STAT1 signaling homeostasis. Based on the crystal structure of pSTAT1, there is a direct polar contact between N574 and K511, providing a possible hypothesis that the N574I mutation could disturb STAT1 ubiquitination at position K511. However, mutating both sites did not affect the IFNγ induced pSTAT1-level nor expression of the IFNγ stimulated gene *Ifit1*.

Our work adds to the general knowledge of STAT1 as a transcription factor corroborating its homodimeric state in resting conditions and potential to form tetramers upon stimulation to drive transcription (32, 38). We also demonstrate that the nuclear mobility can be negatively correlated with an enhanced activity of a transcription factor, as previously described for the glucocorticoid receptor, another transcription factor that becomes translocated to the nucleus upon stimulation (57). However, this correlation is not always present as shown by the R274W mutant, demonstrating enhanced activity as transcription factor while not being significantly slower than WT in the nucleus. Next, there seems to be no significant trend in the correlation between average STAT1 stoichiometry and average transcriptional activity of STAT1, as represented in Supplementary Figures 4C–F.

Our study has several limitations. STAT1 molecules are tagged with GFP, which might affect cellular dynamics, although WT STAT1-GFP was used as reference in all experiments. Next, extrapolation from U3A and HeLa cells toward PBMCs or monocytes remains uncertain, but our live-cell imaging assays require robust cellular models and fluorescently tagged proteins. Currently, we cannot reliably correlate the molecular mechanisms, the clinical phenotypes and/or response to ruxolitinib treatment, due to the small number of mutations analyzed and due to the small number of patients reported to date bearing each individual mutation (30 for R274W, 3 for R321S, 1 for N574I, and 2 for T419R). Far from being an exhaustive description of all STAT1 GOF mutants and potential molecular mechanisms, this study aimed to contribute toward a better and precise molecular understanding of STAT1 GOF and STAT1 molecular behavior in general. Ultimately, if more STAT1 GOF mutations could be tested in the life-imaging assays, specific cellular phenotypes could possibly be linked with clinical presentations and help in stratifying patients for specific treatments.

In conclusion, we identified three distinct molecular mechanisms that alter the normal STAT1 activation/deactivation cycle, and result in a STAT1 GOF phenotype (increased pSTAT and stimulation of ISG), depending on the specific mutation: (1) a faster accumulation in the nucleus, (2) a near-immobilization in the nucleus, and (3) a reduced nuclear mobility together with or due to a decreased dephosphorylation. How and whether these distinct molecular mechanisms might contribute to the phenotypic heterogeneity and responsiveness to JAK inhibitors is still unknown. Future work is necessary to disentangle this relation.

## Data Availability Statement

The datasets generated for this study are available on request to the corresponding author.

## Author Contributions

RS conceived the original idea. SG carried out the experiments and analyzed the data. JH designed the RICS analysis. VL set up and provided technical help for the RICS measurements. VL and JH contributed to the interpretation of RICS results. RS, RG, and SG contributed to the interpretation of the results. RG and RS supervised the project. SG, RG, and RS wrote the manuscript with input from all the authors.

## Conflict of Interest

The authors declare that the research was conducted in the absence of any commercial or financial relationships that could be construed as a potential conflict of interest.
