# Supplementary material for: Live Cell Imaging Demonstrates Multiple Routes Toward a STAT1 Gain-of-Function Phenotype
Source: Front Immunol. 2020 Jun 9;11:1114. doi: 10.3389/fimmu.2020.01114 (PMC7296103; doi:10.3389/fimmu.2020.01114)
Supplement: Supplementary file 1 [file Data_Sheet_1.pdf]

Supplementary figure 1

U3A cells

A

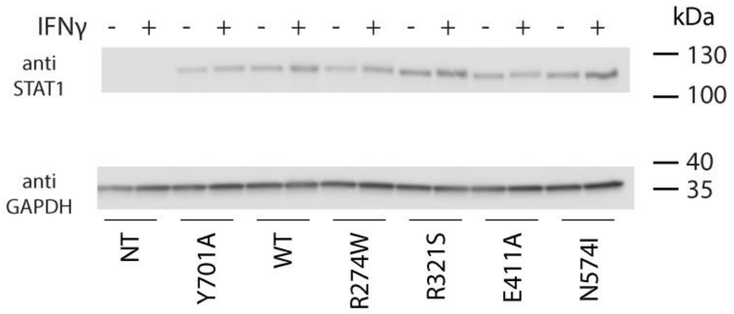

Supplementary figure 2

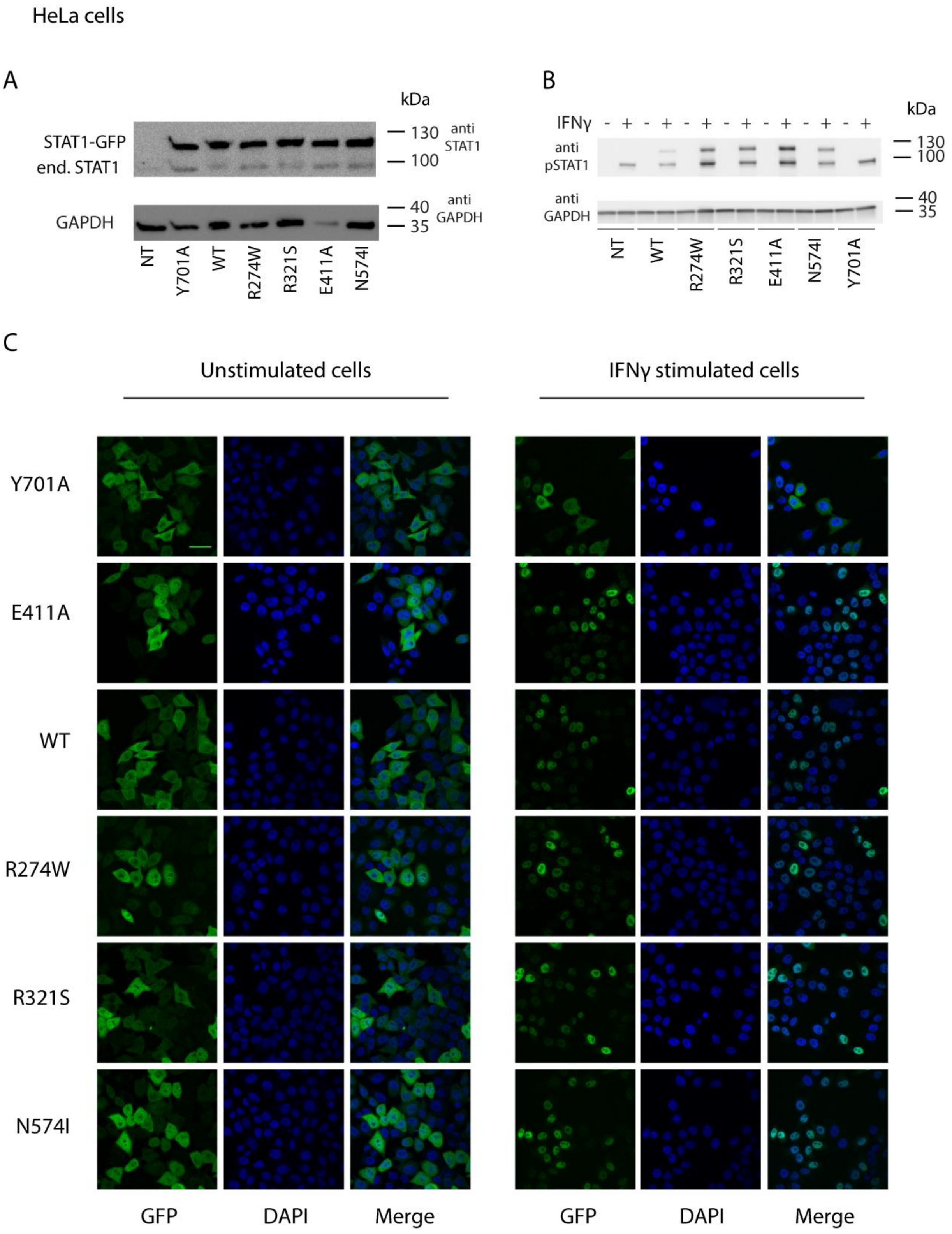

Supplementary Figure 3

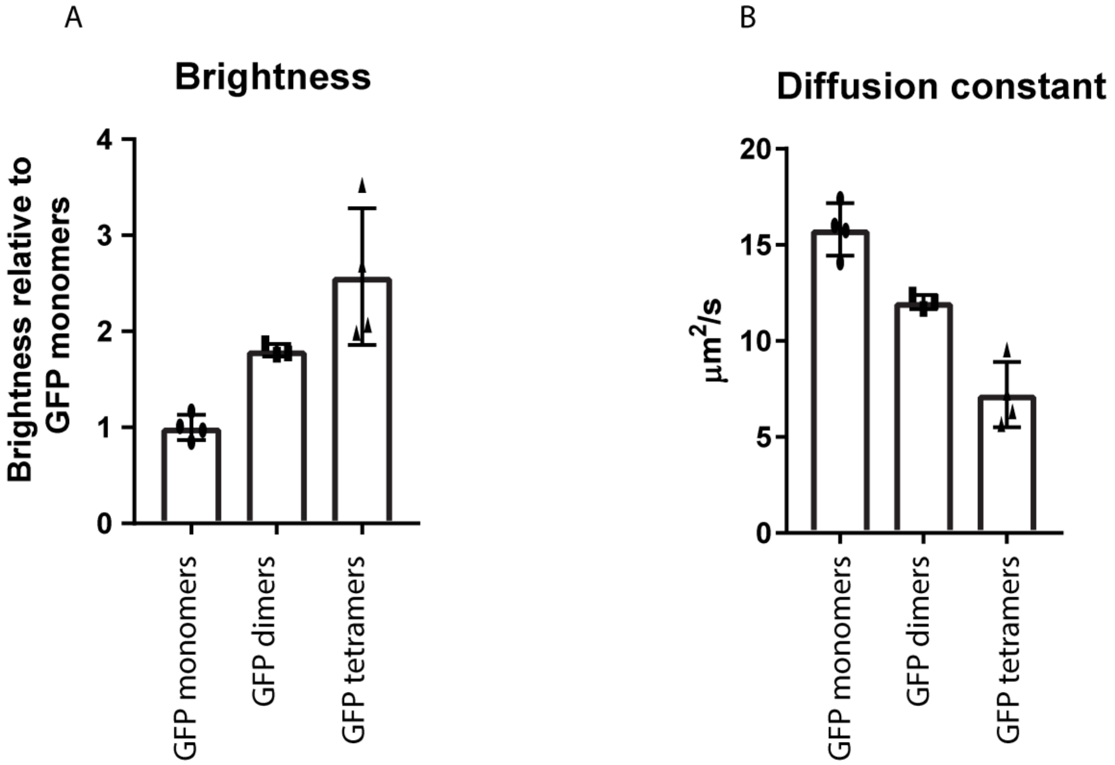

Supplementary Figure 4

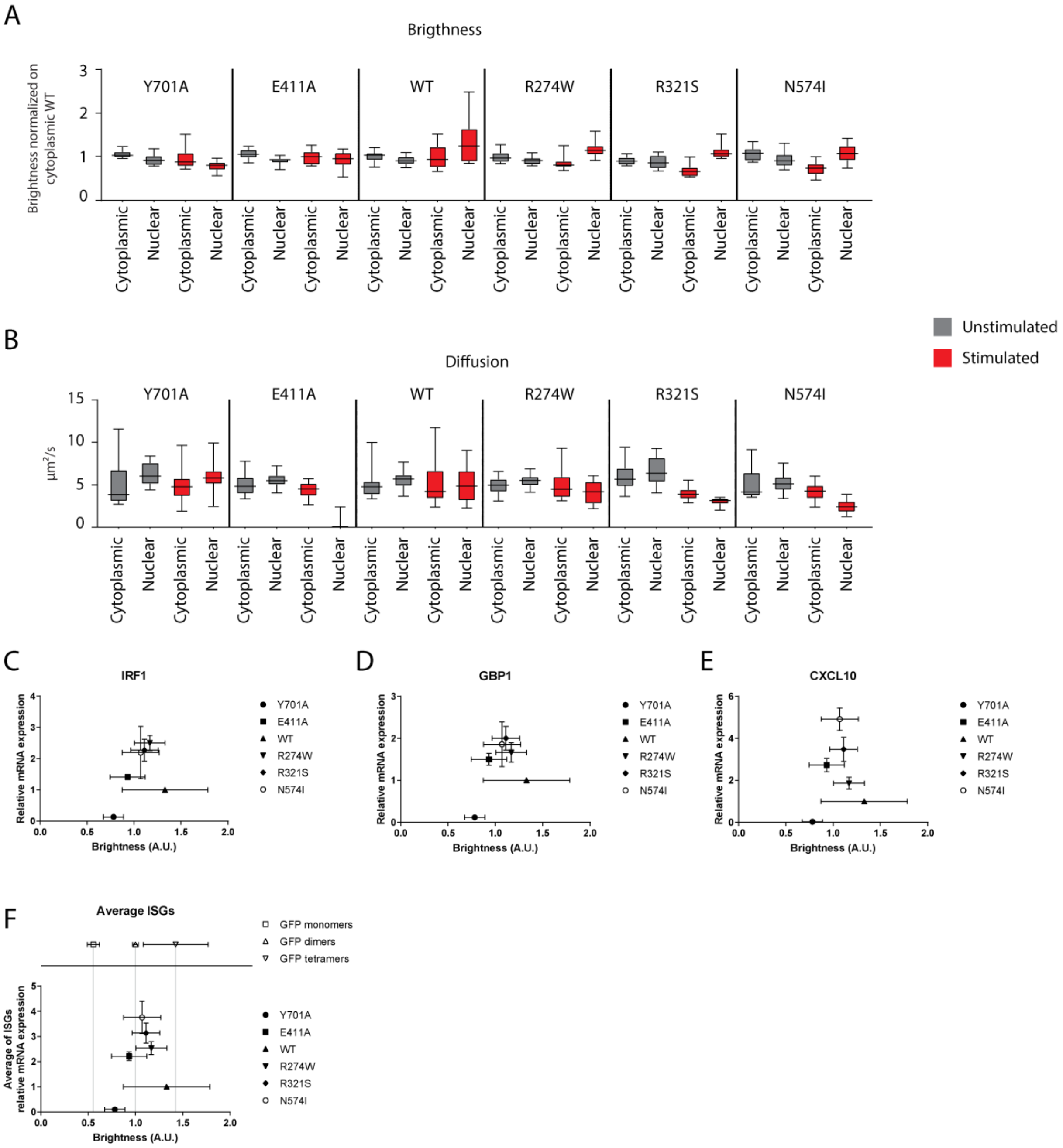

Supplementary figure 5

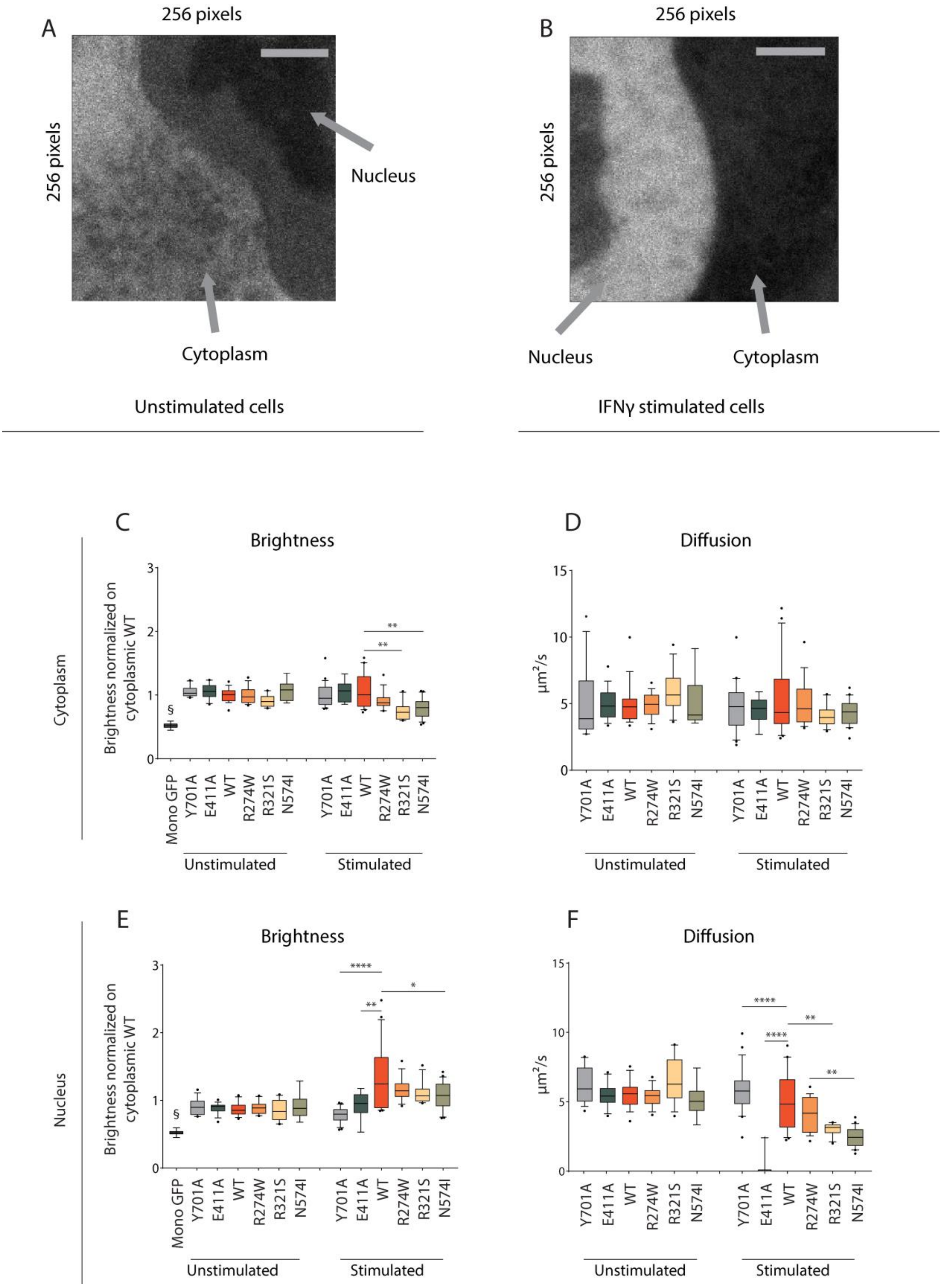

Supplementary Figure 6

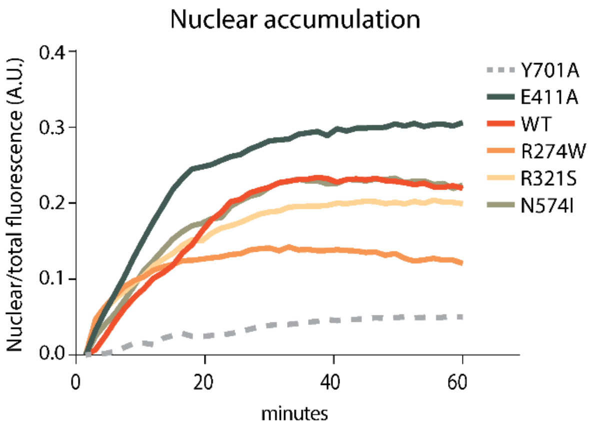

Supplementary figures legends:

Supplementary Figure 1:

Characterization of U3A cell lines. Western Blots of whole cell lysates from U3A cell lysate, stained with anti-STAT1 and anti-GAPDH.

Supplementary Figure 2:

Characterization of HeLa P4 STAT1 expressing cell lines. A) Western blots of whole cell lysates from HeLa P4 cell lysate, stained with anti-STAT1 and anti-GAPDH. B) Western blot of HeLa P4 cell lysate stained with anti-pSTAT1 and anti-GAPDH. Cells were stimulated for 1h with IFNγ (1U/µl) or left unstimulated. C) STAT1 re-localization after IFNγ stimulus. Left panel: unstimulated HeLa P4 cells. Right panel: HeLa P4 cells after 1h of IFNγ (1U/µl) stimulation. Endogenous level of STAT1 in non-transduced cells was lower than the endogenous level of STAT1 in STAT1 WT and STAT1 GOF transduced cells, possibly due to the positive feedback that STAT1 has on STAT1 expression(Yuasa and Hijikata, 2016).

Supplementary Figure 3:

Brightness (A) and diffusion constant (B) measured with RICS of GFP monomers, GFP-GFP fusions (dimers) and GFP-GFP-GFP-GFP fusions (tetramers), transiently expressed in HEK cells. Brightness was normalized on GFP monomers, where absolute photon counts for GFP monomers, dimers and tetramers were, respectively, 9.5 ± 1.1 kHz, 17.2 ± 0.5 kHz and 24.4 ± 5.9 kHz. We used these data to extrapolate STAT1 stoichiometry: the average brightness of unstimulated STAT1-GFP was the double of GFP monomers and in line with the brightness of GFP dimers, corroborating with the general idea that in resting condition STAT1-GFP is present as a homodimer in the cytoplasm. A further increase in STAT1-GFP average brightness, after IFNγ stimulation, indicated that part of STAT1-GFP formed higher order oligomers (tetramers) in the nucleus.

Supplementary Figure 4:

RICS analysis of STAT1-GFP WT and GOF mutations in U3A cells, extra comparisons and correlations. A,B) Average brightness and average diffusion constant measurement of cytoplasmic and nuclear STAT1-GFP fusions in unstimulated U3A cells and cells stimulated with IFNγ (1U/µl). Brightness is expressed in arbitrary units and plotted as fold increase compared to cytoplasmic WT. Data (n>7) are represented in box and whiskers plot. The central line represents the median, while the box extends from the 25th to 75th percentiles with whiskers spanning min-max intervals. Statistical analysis was performed comparing the unstimulated condition to the respective stimulated condition, either in the cytoplasm or in the nucleus: One-way ANOVA multiple pair wise comparisons with Sidak's post hoc test. This figure uses the same data as Figure 5 and Supplementary Figure 5, represented in a different order to show different comparisons. C-E) Expression level of *IRF1*, *GBP1* and *CXCL10* after

IFNγ stimulation (same data as Figure 3) plotted against the average nuclear brightness (after IFNγ) of each respective sample (same data as Figure 5B). F) Average expression of all ISGs tested is plotted against the average nuclear brightness (after IFNγ) of each respective sample. Monomeric, dimeric and tetrameric GFP brightness (same data as Supplementary Figure 3) is plotted on the top of the graph as a reference. Linear regression could not reveal a correlation (R^2 = 0.08, p-value = 0.59 including WT and the different mutants). Since Y701A does not translocate to the nucleus upon IFNγ stimulation, it can be considered as irrelevant for correlating the induction of ISGs and nuclear brightness. However, excluding Y701A from this analysis did not significantly change this result (R^2 = 0.3, p-value = 0.34). Data are presented as average ± standard deviation.

Supplementary Figure 5:

RICS analysis of STAT1-GFP WT and GOF mutations in U3A cells. Representative average of 10 frames of an unstimulated (A) and IFNγ stimulated (B) cell used for RICS analysis. Arrows indicate the nucleus and the cytoplasm of the cells. Gray scale bar is 3.2µm. Average brightness (C,D) and average diffusion constant (E,F) measurement of cytoplasmic and nuclear STAT1-GFP fusions in unstimulated U3A cells (C,E) and cells stimulated with IFNγ (1U/µl) (D,F). § GFP monomers, measured in HEK cells. Brightness is expressed in arbitrary units and plotted as fold increase compared to cytoplasmic WT. Data in panels A-D (n>7) are represented in box and whiskers plot. The central line represents the median, while the box extends from the 25th to 75th percentiles with whiskers spanning 10-90% intervals. Dots outside these intervals are represented as single dots. Statistical analysis: One-way ANOVA with Tukey's post hoc test.

Supplementary Figure 6:

STAT1-GFP nuclear accumulation over time for the respective STAT1 cell lines following IFNγ stimulation. A) Representative experiment (n=4) for nuclear accumulation of fluorescent GFP signal over a 60 min time-lapse in stable STAT1-GFP expressing HeLa cells. Non-normalized data.
